# Pediatric caregiver involvement in the assessment of physicians

**DOI:** 10.1186/s12909-015-0402-6

**Published:** 2015-08-01

**Authors:** Katherine A. Moreau, Catherine M. Pound, Kaylee Eady

**Affiliations:** 1 Children’s Hospital of Eastern Ontario Research Institute, 401 Smyth Road, Ottawa, ON Canada; 2 Department of Pediatrics, University of Ottawa, 401 Smyth Road, Ottawa, ON Canada; 3 School of Rehabilitation Sciences, Faculty of Health Sciences, University of Ottawa, 451 Smyth Road, Ottawa, ON Canada

**Keywords:** Physician assessment, Caregivers, Parents, Pediatrics, Family-centred

## Abstract

**Background:**

Given the growth and benefits of consumerist and family-centred approaches to pediatric health care, there is a need to involve pediatric caregivers in the assessment of their children’s physicians.

**Discussion:**

We present interconnected questions that are important to address in order to facilitate pediatric caregiver involvement in the assessment of their children’s physicians.

**Summary:**

Pediatric caregivers can be valuable assessors of physicians’ non-technical skills. It is important to conduct additional research on caregiver involvement in assessment activities and create a reflective discourse on this topic. To ensure that pediatric caregivers’ assessments of physicians are formally recognized and advantageous, it is important to understand: (a) what pediatric caregivers can assess; (b) what assessment tools exist for pediatric caregivers; (c) how to create appropriate assessment tools for pediatric caregivers; (d) how to collect pediatric caregivers’ assessments; (e) how to increase the legitimacy, use, and effectiveness of pediatric caregivers’ assessments; and (f) the consequences of pediatric caregiver assessment.

## Background

With the rise of consumerist and family-centred approaches to pediatric health care, physicians must be accountable and responsive to children (i.e., patients under 13 years of age) and their caregivers (i.e., adults who are the patients’ parents or guardians). In pediatrics, physicians must establish positive rapport with patients and their caregivers, provide quality care, and facilitate patients and caregivers’ understandings of medical conditions and management plans. Given the complexities of the pediatric context and the need to demonstrate accountability and responsiveness in care interactions, there is recognition that caregivers can assess the skills of their children’s physicians [1].

Feedback provided from these assessments can in turn be a stimulus for learning and encourage physicians to reflect on and improve their skills. However, to participate fully in the assessment process, caregivers need tools that they can use; tools that acknowledge the intricacies of the pediatric context including, physician-patient-caregiver interactions. While researchers note that family members are potential participants in multisource feedback (MSF), a form of assessment that involves the collection of data on physicians’ performances from two or more raters [2], few assessment instruments have been developed specifically for use by caregivers of pediatric patients. There is also a paucity of literature on the acceptability and feasibility of this form of assessment across different pediatric contexts.

Although the literature on the benefits of engaging pediatric caregivers in physician assessment and education is limited, studies on adult patient involvement have shown that they enjoy it, experience increased self-confidence, and view their educative roles as therapeutic [3–5]. Adult patients have also reported deeper understandings of their health, the health care system, and physician-patient relationships [6, 7]. Moreover, Burford et al. [8] found, in their exploration of physicians’ and patients’ perceptions of patient feedback, that physicians responded positively to this form of feedback and acknowledged its educational value for improving their practices. Adult patients in this study also described how the opportunity to provide assessments of their physicians empowered them, gave them a voice, and validated their opinions [8]. In addition, researchers have shown that when physicians receive negative feedback from patients, they are likely to modify their behaviours because they see these individuals as credible and in positions to witness their actions in real time [9].

Yet, regardless of these reported benefits, many physicians, especially those in pediatric contexts, do not formally or routinely obtain and use caregiver assessment data. The lack of progress in this area is in large part attributable to the minimal amount of research on both how to develop pediatric caregiver assessments tools and how to involve caregivers in the assessment of their children’s physicians. In recognition of this void, this paper presents interconnected questions that are important to address in order to facilitate pediatric caregiver involvement in the assessment of their children’s physicians. These thought provoking questions focus on: (a) what pediatric caregivers can assess; (b) what assessment tools exist for pediatric caregivers; (c) how to create appropriate assessment tools for pediatric caregivers; (d) how to collect pediatric caregivers’ assessments; (e) how to increase the legitimacy, use, and effectiveness of pediatric caregivers’ assessments; and (f) how to understand the consequences of pediatric caregiver assessment. By offering potential answers to these questions, we hope to generate discussion, debates, and additional research on this evolving area of assessment.

## Discussion

### What can pediatric caregivers assess?

To facilitate pediatric caregiver involvement in the assessment of physicians, it is important to consider what they experience and observe in their interactions with physicians and thereby can assess. Although there are many technical elements of physicians’ performances that pediatric caregivers cannot assess, we argue that there is a wide range of non-technical skills that they can assess. Often referred to as physicians’ cognitive and social abilities, these non-technical skills contribute to safe, effective, and efficient care. Since pediatric caregivers are often at their children’s bedsides, they are ideally positioned to assess these non-technical skills, which can encompass, for example, physicians’ communication, professionalism, advocacy, and situational awareness. Assessment of these skills by caregivers is valuable because they have first-hand experience of how physicians interact with them and their children and thus, can bring different perspectives to assessment processes [3, 10, 11].

### What assessment tools exist for pediatric caregivers?

To facilitate pediatric caregivers’ involvement in the assessment of their children’s physicians, it is also important to be aware of the existing assessment tools developed for pediatric caregivers. However, our review of the literature shows that very few assessment tools have been developed specifically for pediatric caregivers. Boon and Steward [12], in their review of 44 patient communication assessment tools (i.e., tools used in all contexts including, adult and pediatric medicine) only found one which had undergone some form of validity and reliability testing and involved parents of pediatric patients in the assessment process. This tool, by Street [13], includes 14 Likert items to obtain ratings from parents on physicians’ abilities to communicate, show interpersonal sensitivity, and build partnerships with patients and their families.

Similarly, Chisholm and Askham [14] in their review of questionnaires designed to gather feedback from patients on individual physicians, found only one tool designed for use in pediatrics, the Sheffield Patient Assessment Tool (SHEFFPAT) [10, 15], which met their standards for validity and reliability testing. The SHEFFPAT includes 13 items as well as sections for patient characteristics and general comments. It focuses on the quality of physician consultations, parental understanding of the patient’s condition and its treatment, confidence in self-care, and the physician’s interpersonal and confidentiality skills. Crossley and Davies [15] used a rigorous process, which included a review of the literature and a nominal consensus activity with pediatricians on the key components of a good pediatric consultation, to develop the SHEFFPAT. They also piloted the instrument to ensure that the items, rating scales, and layout were comprehensive, comprehensible, and acceptable for parents [10]. Moreover, studies have been completed to establish the internal consistency, factor structure, physician-level reliability, criterion validity, and construct validity of the SHEFFPAT [10, 16]. To date, the SHEFFPAT has been used to assess physicians in a variety of pediatric settings [17]. McGraw et al. [1] later modified this tool and renamed it the Paediatric Carers of Children Feedback tool (PaedCCF). This 17-item tool includes 12 of the items from the SHEFFPAT and five additional items that relate to physicians’ skills to communicate treatment/condition risks and their ability to help caregivers access additional support– items that were determined to be missing on the original SHEPPAT [14]. McGraw and her team [1] have evaluated the reliability, validity, feasibility, and acceptability of using the PaedCCF across multiple pediatric settings and reported favorable results.

Although the above-mentioned tools are of high quality and are well suited for pediatric caregivers to assess their children’s physicians, they may not be relevant to all pediatric contexts or for all assessment purposes. Thus, there is a strong justification for additional testing of the above-mentioned assessment tools as well as the creation of new ones. It is important to ensure that the tools are sound, able to provide relevant and accurate assessment scores of physicians’ skills, and appropriate for the context(s) and purposes for which they will be used.

### How can we create appropriate assessment tools for pediatric caregivers?

When creating assessment tools that pediatric caregivers can use to assess their children’s physicians, it is important to engage in activities that focus on the involvement of caregivers in the creation process. By involving caregivers, the relevance of the tools as well as the validity and reliability of the assessment scores and the interpretations generated from them can improve. While the Picker Institute emphasizes that individuals who are developing assessment tools should ask patients/caregivers what physicians’ skills they feel confident in assessing, they also note that this step rarely happens [14]. This is unfortunate because when given the opportunity, caregivers can ensure that the design of the assessment tool, including the length and wording, is conducive to them and will facilitate their accurate completion of it. Caregivers can also identify specific design flaws, deficiencies, or potential problems with the tool that researchers may not. Moreover, if caregivers are engaged in the development phase, it is probable that they will use the tool more actively in future and promote its use to others.

There are various ways to involve caregivers in the creation of assessment tools. For example, focus groups with caregivers can be used to seek out their thoughts on potential items, formatting, identifiable behaviors that align with the physicians’ skills, abilities, or competencies that the tool will measure, or relevant and observable activities in physician-patient-caregiver interactions. This information should then be used in conjunction with other items found in the literature or existing measures designed to assess physicians’ skills. Once a draft assessment tool is developed, researchers can also reach out to caregivers who, for example, are members of their hospital’s family advisory committee or the targeted caregiver population to review the draft tool. When using this approach, caregivers can rank each item for inclusion (e.g., most definitely include this item, include this item, possibly include this item, definitely do not include this item) as well as indicate whether or not they experience each item in their interactions with their children’s physicians. Based on the findings of this review, informed decisions can be made about which items to keep or revise on the assessment tool.

Another technique to engage caregivers in the creation of assessment tools is cognitive interviewing. Cognitive interviewing can be used to explore the ways in which caregivers understand, mentally process, and respond to the items on draft assessment tools [18]. Through this activity, assessment tool developers can ensure that the tool measures what they intended, that caregivers understand and interpret items correctly, and that sources of response errors in the tool are minimal [18].

There are two main approaches to cognitive interviewing: think-aloud and verbal probing [18, 19]. Think-aloud is an activity where caregivers verbalize their thought processes as they respond to the items on an assessment tool. The interviewer plays a passive role in the think-aloud process. The interviewer reads each item on the assessment tool to the caregiver and records the processes that the caregiver uses to arrive at his/her response to the item. Conversely, with verbal probing the interviewer reads each item on an assessment tool to the caregiver, records their response, and uses either retrospective or concurrent probing to solicit information on the items and basis for the caregiver’s response. With retrospective probing, the caregiver answers questions about the tool in the form of a debriefing session after he/she has reviewed and responded to the entire tool. Whereas with concurrent probing the interviewer asks the caregiver a series of questions after they review and answer each item. Possible questions posed might include the following: (1) Can you explain to me in your own words what this item is asking you? (2) Can you explain to me why you chose the answer that you did? (3) Do you feel that the response options allowed you to answer the item appropriately? (4) Do you find the item easy or difficult to answer? [18].

Although researchers have identified that think-aloud or verbal probing is integral for tool design, they are not widely used [20]. This lack of use is problematic. Ultimately, the findings from these cognitive interviewing techniques can be used to: (a) enrich the definitions or understanding of specific items, (b) understand and document differences in item interpretations across sub-groups of caregivers, and (c) revise or delete items with extreme differences in interpretation, including wide deviations among individual caregivers or large inconsistencies with the intended interpretations of the items.

### How can we collect pediatric caregivers’ assessments?

One way of collecting pediatric caregivers’ assessments of their children’s physicians is through technology. In recent years, many organizations have transitioned away from pencil-and-paper questionnaires to electronic ones. Penny [21] compared the use of these two forms of questionnaires and concluded that the method did not influence the responses given or the consistency of the raters. In fact, Penny [21] concluded that the use of electronic questionnaires for assessment and feedback provides an economy and immediacy to the assessment process that is beneficial for all those involved. Moreover, given the prevalent use of, for instance, cellphones, iPads, social media, and instant messaging, it is easy to see that technology is a seamless, natural part of everyday life and that we are living in a digital age. In fact, Statistics Canada [22] found that eight out of ten households had access to the Internet and that over one-half of connected households used more than one type of electronic device to connect to the Internet. As such, technology fluency presents a unique opportunity to engage pediatric caregivers in physician assessment and, as such, should become an instrumental aspect of the collection of caregivers’ assessment data.

### How can we increase the legitimacy, use, and effectiveness of pediatric caregivers’ assessments?

In order to increase the legitimacy, use, and effectiveness of caregivers’ assessments of physicians, relevant stakeholder groups including caregivers, physicians, and hospital administrators must be aware of the existence and value of these assessments. Stakeholders need training in terms of why this form of assessment is important, the purposes and intended use of it, as well as in the development and utilization of caregiver-specific assessment strategies and tools [23]. Drawing from the MSF literature, researchers have shown that the implementation of any assessment system requires communication among all those involved. Communication throughout the assessment process helps demystify the approach, provides the basis for increased involvement among relevant stakeholders, and is especially important once assessment results are collected and returned for stakeholder reflection and utilization [23]. Faculty development opportunities, training sessions, caregiver champions who advocate for and explain this approach to other caregivers, as well as signage throughout organizations that promote caregiver engagement in the assessment of physicians are all important options to consider in order to strengthen the capacity and use of caregiver assessments within different pediatric contexts.

### How can we understand the consequences of pediatric caregiver assessment?

Lastly, caregivers’ assessments of their children’s physicians will have consequences on learning, teaching, and assessment practices of physicians, continuing professional development (CPD)-educators, and caregivers and hopefully impact clinical practices and patient outcomes. These consequences can be intended or unintended as well as positive or negative [24]. In order to understand these consequences, it is important for physicians, CPD-educators, caregivers, and other relevant stakeholder groups to receive feedback on the assessment processes and have opportunities to reflect on their active involvement in assessment activities. This feedback and reflection can range in structure, format, and content and depend on the needs and requirements of the various stakeholder groups. For instance, researchers can interview or survey the various stakeholders after the given assessment activities. This would allow them to determine stakeholders’ thoughts on the assessment processes and explore how the assessment data or activities influenced them or the children that they care for.

## Summary

Pediatric caregivers can be valuable assessors of physicians’ non-technical skills. To ensure that pediatric caregivers’ assessments of physicians are formally recognized and advantageous, it is important to understand: (a) what pediatric caregivers can assess; (b) what assessment tools exist for pediatric caregivers; (c) how to create appropriate assessment tools for pediatric caregivers; (d) how to collect pediatric caregivers’ assessments; (e) how to increase the legitimacy, use, and effectiveness of pediatric caregivers’ assessments; and (f) the consequences of pediatric caregiver assessment. By conducting additional research in this area and investing in activities to ensure that caregiver assessment tools are appropriately developed and used, we will be able to receive first-hand accounts and unique information on pediatric caregivers’ perceptions of physicians’ non-technical skills. This data can be used for decision-making, the development of educational initiatives, self-improvement, or the enhancement of health care delivery. To improve stakeholders’ understanding and involvement of caregivers in the assessment of physicians, there is a need for further discourse and research.

Since minimal discourse and research exists on the best ways to involve pediatric caregivers in physician assessment, it is beneficial to further debate, discuss, and reflect on this topic. Possible questions to stimulate this deliberation include the following: What roles do pediatric caregivers want to play in the assessment of their children’s physicians? How do physicians react to and use caregivers’ assessments of their non-technical skills? What strategies support caregivers’ involvement in the assessment of their children’s physicians? How can technology facilitate pediatric caregiver involvement in the assessment of physicians? What are the strengths and limitations of using various types of technology for caregiver assessment? Ultimately, by answering these questions as well as documenting and reflecting on pediatric caregiver assessment activities, we can contribute to the literature on the assessment of physicians and build on the very limited body of empirical research on pediatric caregiver involvement in physician assessment.
